# Initial evaluation of a practical PET respiratory motion correction method in clinical simultaneous PET/MRI

**DOI:** 10.1186/2197-7364-1-S1-A40

**Published:** 2014-07-29

**Authors:** Richard Manber, Kris Thielemans, Brian Hutton, Anna Barnes, Sebastien Ourselin, Simon Arridge, Celia O’Meara, David Atkinson

**Affiliations:** University College London, London, UK; University College Hospital, London, UK

Respiratory motion during PET acquisitions can cause image artefacts, with sharpness and tracer quantification adversely affected due to count ‘smearing’. Motion correction by registration of PET gates becomes increasingly difficult with shorter scan times and less counts. The advent of simultaneous PET/MRI scanners allows the use of high spatial resolution MRI to capture motion states during respiration [1, 2]. In this work, we use a respiratory signal derived from the PET list-mode data [3, 4], with no requirement for an external device or MR sequence modifications.

**Figure 1:**
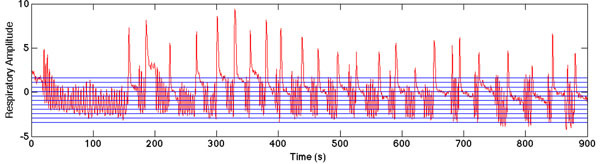
Respiratory signal throughout clinical 15 minute PET acquisition, including ‘free breathing’ and ‘breath-hold’ sections. Horizontal lines used to bin the PET data based on signal amplitude are also shown. Deep inhalation periods are excluded.

**Figure 2:**
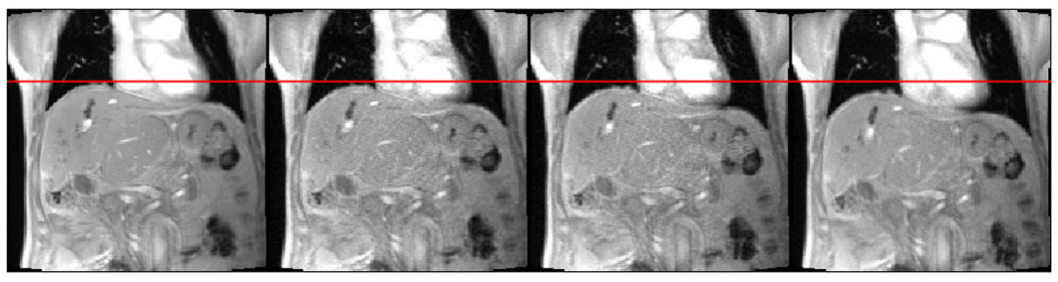
Binned and averaged MRI slices for 4 out of the 10 bins; ranging from end-expiration to end-inspiration.

**Figure 3:**
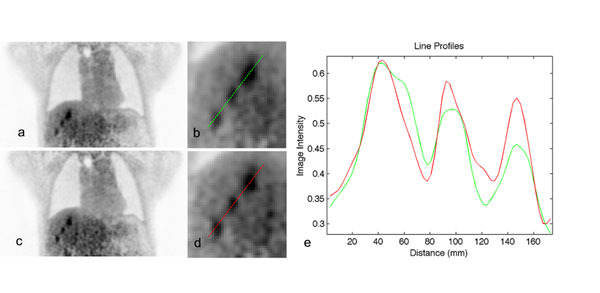
Uncorrected image (a) with ROI (b), motion-corrected image (c) with ROI (d), line profiles through the liver lesions in both images (e). Image intensity scale is in arbitrary units.

Clinical PET data are grouped into 10 respiratory bins based on respiratory signal amplitude derived from the PET list-mode data (Deep breaths outside defined limits are ignored) (Figure 1). During an extra post-scan 30s PET/MRI acquisition, rapid 2D Gradient Echo MR images are collected and grouped into these 10 respiratory bins. Images in each bin are averaged to form one image per bin (Figure 2), which are registered to a reference image, forming a patient-specific motion model. Motion estimates from the model are applied directly within the reconstruction of the clinical PET list-mode data using Motion Compensated Image Reconstruction (MCIR) [5], to form one motion-corrected image. On two human subjects (^18^F-FDG - 1 multiple liver lesions, 1 cardiac) we present PET data motion-corrected with an MRI motion model. Images are assessed visually, with line profiles through ROIs (Figure 3), and by change of pixel intensity in regions of high activity.

In the liver case we see a decrease in tumor ‘smearing’ after MRI model-based correction (Figure 3). Other areas of high activity in the liver, only marginally visible in the uncorrected image, become apparent in the motion-corrected image. Average intensity increase over the 3 lesions is 11%, while increase in intensity in the cardiac wall in the cardiac patient is 10%.
